# The Protective and Regenerative Potential of Lactoferrin in Hair and Skin Health

**DOI:** 10.3390/ijms27104451

**Published:** 2026-05-15

**Authors:** Nicole Kaplan, Giorgio Dell’Acqua

**Affiliations:** 1 Helaina Inc., New York, NY 10010, USA; 2 Dellacqua Consulting, Jersey City, NJ 07302, USA; giorgio@dellacqua-consulting.com

**Keywords:** Lactoferrin (Lf), dermal papilla (DP) cells, iron homeostasis, oxidative stress, wound healing, hair follicle cycling, skin barrier function

## Abstract

Lactoferrin is a naturally occurring bioactive glycoprotein that is part of the body’s innate immune system and has essential roles in iron metabolism, microbial defense, inflammation regulation, and tissue repair. It supports the natural regulation of iron bioavailability in skin and hair follicles, helping to reduce excess free-iron-driven oxidative stress while preserving levels of necessary iron for cellular functions. Lactoferrin promotes cell regeneration by increasing proliferation across in vitro systems, stimulating wound healing in scratch assays, and boosting matrix production in fibroblast models. Lactoferrin can also modulate inflammatory signaling involved in skin and hair physiology by providing balanced cytokine suppression, suggesting potential value in cosmetic or dermatological applications. Here, we present the first focused summary of lactoferrin’s role specifically in skin and hair biology, distinguished from prior reviews in systemic or multi-system broad health contexts. We link mechanistic insights with clinical and preclinical evidence and uniquely map molecular functions to dermatologic and trichologic outcomes. We also provide an overview of clinical skin studies that have explored lactoferrin as a supportive agent in conditions such as acne, and highlight that, despite mechanistic plausibility, there are no existing available reports of well-controlled human clinical trials leveraging lactoferrin for hair-focused outcomes. In summary, we propose lactoferrin as not just an anti-inflammatory molecule, but also as a microenvironment stabilizer, and particularly relevant for hair and skin support as an alternative to pharmacological interventions. By addressing both established and underexplored applications, this review provides a translational framework for clinical development and provides a comprehensive rationale behind leveraging lactoferrin for hair and skin epithelial health.

## 1. Introduction

Lactoferrin is a multifunctional, iron-binding glycoprotein that has protective and tissue healing capacities throughout the life of an organism [1]. It is present in exocrine secretions—including milk, saliva, and tears—and is released during inflammation by neutrophils and synthesized by epithelial cells. Structurally, lactoferrin is an ~80 kDa protein belonging to the transferrin family, composed of N- and C-terminal lobes, and connected by a flexible linker. Lactoferrin’s capacity to fight oxidative stress, reduce inflammation, and promote immune balance has been reported in many studies [1,2,3]. The structural heterogeneity of the protein arises in part from its ability to be differentially modified by phosphorylation, acetylation, glycosylation, and other post-translational modifications [4]. This post-translational diversity enables lactoferrin to have different binding interactions, biological activities, and downstream effects. Thus, lactoferrin’s structural heterogeneity is instrumental in its diverse bioactivities and functional capabilities [1,4].

Lactoferrin’s structural and functional flexibility is further highlighted when lactoferrin is processed at sites of inflammation (specifically by neutrophil proteases) [5] or during digestion (by gastric enzymes and partially in the duodenum) [6], delivering functional peptides with different active domains. These peptides have been associated with immune regulation, anti-inflammatory, and antimicrobial properties [7,8], possibly modulating the gut microbiome [9] and exhibiting systemic activity to mitigate microbial infections [10].

**Figure 1:**
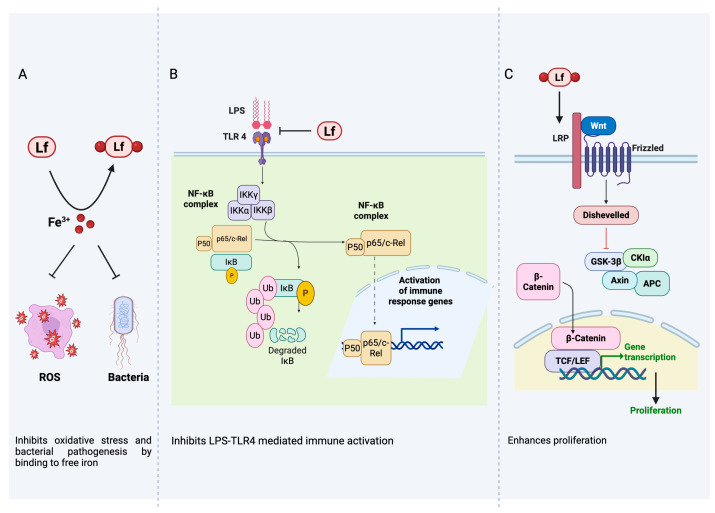
Mechanisms of action of lactoferrin: (**A**) Lactoferrin (Lf) by binding to free iron (Fe^3+^), limits the availability of iron to pathogens and mitigates oxidative stress, contributing to antimicrobial and antioxidant activity; (**B**) By binding to TLR4 ligands/agonists such as LPS, lactoferrin inhibits NF-kB downstream signaling and inhibits the production of pro-inflammatory cytokines; (**C**) By upregulating β-catenin and activating the Wnt signaling pathway, lactoferrin enhances cell proliferation and promotes tissue regeneration during wound healing. Created with BioRender.com.

The chemistry of lactoferrin is complex, allowing lactoferrin to act multifunctionally, where different forms and structures of the protein may be more effective. Furthermore, the structural flexibility of lactoferrin enables multiple mechanisms of action (MoA) and engagement of various signaling pathways (Figure 1). Lactoferrin is well recognized for its strong binding capacity to free iron and its ability to reduce oxidative stress [1]. This property not only aids in limiting pathogen activity [11], but also plays an important role in regulating the activity of certain iron-dependent enzymes and proteins involved in protective and regenerative processes [12]. Lactoferrin’s regulation of iron availability also links host defense to oxidative stress control [13]. Free iron catalyzes the formation of reactive oxygen species (ROS) through Fenton chemistry, contributing to oxidative damage during infection and inflammation. By tightly binding free iron, lactoferrin reduces ROS generation and thereby protects cellular components from oxidative damage [14,15,16].

Lactoferrin exhibits context-dependent immunomodulatory activity, characterized by the attenuation of excessive pro-inflammatory signaling and the promotion of immune homeostasis. Lactoferrin and its peptides have been shown to reduce levels of pro-inflammatory cytokines such as TNF-α and IL-6 by suppressing the NF–κB signaling pathway, a central driver of pro-inflammatory responses [7,17]. In a clinical study, lactoferrin supplementation has been linked to a pronounced increase in circulating neutrophil precursors, and an associated reduction in TNF-α and IL-6 production [18]. Although the mechanisms described here are associated with direct immune system regulation, lactoferrin also works through biochemical and cellular mechanisms that make it relevant for protection against environmental stressors such as pathogens and UV radiation [19,20], making it particularly relevant for hair and skin applications. Thus, lactoferrin is not strictly an immune switch that controls activation or suppression per se, but rather is an immune and oxidative balancing factor with potential use in the cosmetics industry.

Previous reviews have discussed the general nutritional, antioxidant, and regulatory properties of lactoferrin across multiple systems and applications [21,22,23]. The present review specifically focuses on hair and skin as the primary biological targets. Its novelty lies in integrating both preclinical and clinical evidence related to dermatological and follicular outcomes, while evaluating lactoferrin within the context of skin and hair health applications.

## 2. The Role of Lactoferrin in Host Defense Against Infection

Lactoferrin’s role in host defense against infection represents its most fundamental biological function and provides a framework for its diverse activities. Its wide distribution in mucosal secretions, as well as its release at sites of infection and inflammation, enables it to contribute to first-line defense against microbial challenge [24,25]. Its antimicrobial activity is mediated through both iron-dependent and iron-independent mechanisms.

The primary antimicrobial function of lactoferrin is its high-affinity sequestration of ferric iron (Fe^3+^), an innate immune process described as nutritional immunity. Iron is a critical requirement for the growth of many microorganisms. By limiting iron availability, lactoferrin restricts microbial growth [24]. In addition to this mechanism, lactoferrin exerts antimicrobial effects through direct interactions with bacterial, viral, and fungal surfaces. It can bind to pathogen-associated molecular patterns such as lipopolysaccharide (LPS) in Gram-negative bacteria, destabilize microbial membranes, and interfere with pathogen adhesion and entry into host cells [24,26]. These activities extend across a broad spectrum of pathogens, including enteric bacteria, respiratory viruses, and opportunistic fungi, highlighting lactoferrin’s role as a non-specific but effective antimicrobial agent.

Beyond direct pathogen control, lactoferrin plays a critical role in shaping host immune responses by simultaneously controlling microbial burden and preventing excessive inflammation, which is essential for maintaining tissue integrity at sites of host–pathogen interaction [3,15]. Lactoferrin’s antioxidant activity complements its antimicrobial and immunomodulatory functions, reinforcing its role in maintaining tissue homeostasis [14].

At epithelial interfaces where microbial exposure is continuous, these functions are highly integrated. Lactoferrin contributes to barrier protection by modulating microbial communities, regulating immune responses, and supporting epithelial integrity [13,26,27]. This integrated role in host defense provides a mechanistic foundation for its broader applications in epithelial systems, including skin and hair.

## 3. Lactoferrin-Mediated Iron Regulation in Skin and Hair Biology

### 3.1. Iron Trafficking in the Skin and Hair Follicle Microenvironment

Immune cells, such as macrophages and activated Natural Killer (NK) cells, are recruited to injured, inflamed, or proliferating skin and hair follicles, where they regulate iron balance and metabolism [28,29,30]. Tissue macrophages regulate iron by recycling it, storing it in ferritin, and releasing it via the iron exporter ferroportin, depending on metabolic need [28,29,30]. Macrophage ferroportin-mediated iron export and release is essential for the proliferation of skin and follicle epithelial cells. A study in mice demonstrated that when ferroportin is knocked out specifically in macrophages, the animals failed to develop hair, highlighting the importance of localized iron trafficking in the hair follicle and the need for balanced iron control. Wound healing following skin damage was also reduced in these mice, suggesting that cellular repair and growth are both very iron-dependent, as healing was delayed when macrophages could not deliver iron [29].

An in vivo study in mice with transferrin deficiency demonstrated that iron is essential for skin development. The study reported that invariant Natural Killer T (iNKT) cells reside in the skin during early life and serve as a source of transferrin by upregulating genes encoding transferrin and transferrin receptor [30]. Upregulation of transferrin, an iron-regulating protein, supported hair growth by promoting the proliferation of hair follicle progenitor cells in these mice. These iNKT cells remain present in later life to support follicle homeostasis, highlighting the important role of iron regulation throughout the full life cycle of the hair follicle to support healthy hair growth [30].

**Figure 2:**
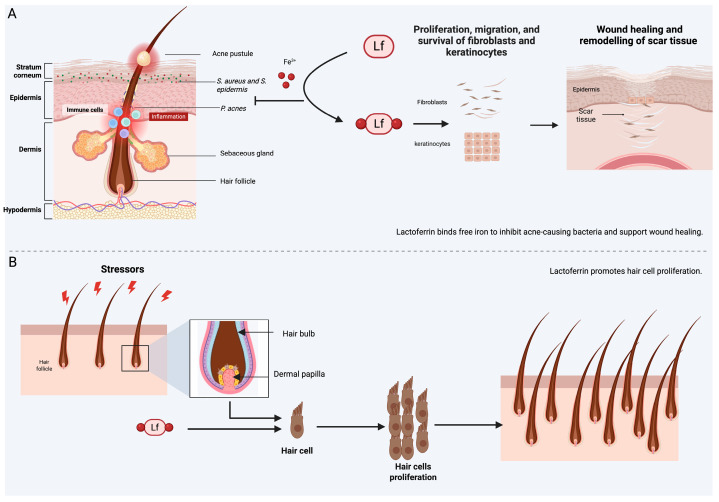
Lactoferrin’s potential in hair and skin applications: (**A**) Lactoferrin (Lf), by binding and sequestering the free iron (Fe^3+^), limits iron availability for acne causing bacteria, also facilitates iron regulation to support the wound healing process by promoting fibroblast and keratinocyte proliferation; (**B**) Lactoferrin promotes the proliferation of hair cells by activating signaling pathways within dermal papilla cells within the hair bulb, enhancing cellular regeneration. Created with BioRender.com.

Iron is an essential co-factor for enzymes involved in DNA replication, DNA repair, [31] and collagen biosynthesis [32]. Iron metabolism in the cell is also essential for oxygenation and energy production; however, iron levels need to be controlled. When in excess, free iron can accelerate oxidative damage in the skin and contribute to skin aging, especially when combined with UVA exposure [33]. Reducing local iron availability in the hair follicular niche can reduce oxidative stress by shifting macrophages to a healing state and suppressing pro-inflammatory signaling [34]. Thus, it is not simply binding versus delivering iron that is biologically relevant but rather the regulated balance of iron (Figure 2). Here, we propose lactoferrin as a highly sophisticated molecule capable of regulating that balance.

### 3.2. Systemic Iron and Hair Loss

As discussed previously, iron serves as an essential cofactor for several enzymes involved in cell replication [35]. Ribonucleotide reductase, a rate-limiting enzyme in DNA synthesis, is one such enzyme that is highly dependent on iron for its activity [36]. In iron-deficient conditions, there is a reduction in the ribonucleotide reductase activity decreasing the DNA synthesis [37].

Ferritin is an iron storage protein, and serum ferritin reflects the iron levels in the body. Thus, serum ferritin is a standard indicator of iron status in hair loss studies. Hair loss is often observed in women with iron deficiency [38], and there also seems to be a link between low serum ferritin level and Telogen Effluvium (TE), a hair loss condition associated with stress [39,40]. However, the association between hair loss and low serum ferritin levels has been debated for many years, and there is no clear consensus on optimal ferritin levels to reduce hair loss, although a range of 40 to 70 ng/dL has been suggested [40].

While the data linking ferritin levels and hair loss are not entirely consistent [39], a recent study on individuals with TE found a significant correlation between TE and low iron levels [41]. This study showed that individuals taking lactoferrin experienced less TE and less hair loss, suggesting a potential link between systemic lactoferrin intake and protection against stress-induced hair loss [41].

While age-related hair loss is chronic and progressive, stress-related disruptions in hair cycling are typically acute and potentially reversible. Aging-associated hair loss is driven in part by inflammaging, cumulative oxidative stress, and impaired iron homeostasis [42,43], whereas stress-related shedding is characterized by transient cytokine surges and oxidative imbalance that may resolve if the follicular environment recovers [44]. Although the downstream pathways underlying these conditions differ, both share upstream contributors, including oxidative stress and inflammation [42,44] or other citations…?). Lactoferrin acts at these shared nodes through modulation of iron availability, redox balance, inflammatory signaling, and cell survival [3,16,25], suggesting a role in regulating the follicular microenvironment across distinct hair loss phenotypes. By targeting these upstream processes, lactoferrin may address both chronic (age-related) and acute (stress-induced) forms of hair loss through systems-level normalization rather than single-pathway activation, warranting further investigation in hair-focused clinical trials [15].

### 3.3. Lactoferrin as an Iron-Binding Protein to Target Iron-Dependent Conditions in Hair and Skin

Lactoferrin, as the name suggests, is an iron-binding protein with a hundred times more affinity to iron than transferrin [45]. Lactoferrin has two lobes, and each lobe of the protein can bind to an iron ion [46]. The capacity of lactoferrin to reversibly bind iron makes this protein key for regulating iron homeostasis in the body, but it also helps prevent iron-dependent pathogens from proliferating [11].

In vitro studies suggest that iron chelators show potential as an intervention strategy in hypoxic and inflammatory microenvironments in the human hair follicle and skin. By reducing excess free iron—an element implicated in hair loss—iron chelators may stimulate hair growth and rejuvenation [47]. In low oxygen conditions, Hypoxia-Inducible Factor-1 (HIF-1) is activated, promoting tissue formation, cell proliferation, and migration. During wound healing, HIF-1 activates processes such as neovascularization, collagen, and elastin production [47,48].

Hair conditions such as Androgenetic Alopecia (AGA) have been linked to reduced oxygenation and vascularization due to a lack of nutrients reaching the hair bulb, where HIF-1 expression was strongly reduced in AGA patients [49]. Studies conducted in hair cells reported that subsequent activation of HIF-1 can stimulate gene activation responsible for cellular growth [49,50]. Indeed, the HIF-1 signaling pathway has been recently identified as one of the main growth-stimulating pathways in the human follicle, together with other signaling pathways [50].

Minoxidil, commonly used to stimulate hair growth, has been shown to inhibit prolyl-hydroxylase-2 (PHD-2), an enzyme responsible for the degradation of HIF-1 through hydroxylation [51]. Inhibition of PHD-2 can therefore activate HIF-1-dependent transcription of genes such as Vascular Endothelial Growth Factor-1 (VEGF-1), crucial for neovascularization and growth during the anagen phase of the hair follicle [52].

Since PHD-2 is highly dependent on iron as a cofactor, iron chelating agents can effectively inhibit PHD-2-dependent degradation of HIF-1 [34]. Thor D et al. [53] tested the above hypothesis in a clinical study using a topically administered iron chelator. After nine months, hair thickness, density and shine improved significantly compared to baseline. Hair shedding was reduced by 66.8% as early as 6 months [53]. The authors followed up with an additional review suggesting that iron chelators have a potential benefit in autoimmune hair conditions such as alopecia areata (AA) when used as an adjuvant [34]. In fact, iron chelators, by creating a mimetic hypoxic environment, seem to stabilize HIF-1 and at the same time stimulate macrophage polarization and cytokine expression. Reduction in intracellular iron can therefore shift macrophages toward an anti-inflammatory M2 phenotype and suppress NF–κB signaling, with consequent reduction in TNF-α and IL-15, key cytokines involved in autoimmune follicular degeneration [34].

Unlike iron chelators such as Deferoxamine (DFO), which primarily functions through direct depletion of bioavailable ferric iron [54], lactoferrin more adaptively regulates iron availability within inflammatory and epithelial environments. Through its reversible iron-binding capacity, lactoferrin could be leveraged as a naturally occurring iron-regulatory protein to support hair loss [34,53] and skin health [47,48].

## 4. Lactoferrin as a Protective and Regenerative Factor in Hair and Skin: Evidence from Cellular and Animal Models

Beyond its iron-dependent mechanisms, lactoferrin activates distinct signaling pathways that drive protective and regenerative responses. In vitro studies demonstrate that lactoferrin, in its various forms, can interact with tissue-specific receptors to induce different physiological responses, including stress protection, wound healing, cellular migration, and regeneration [55,56,57,58].

Stem cell populations within the skin are central to tissue maintenance, regeneration, and the cell cycle. The hair follicle contains multiple stem cell niches, including epithelial, melanocyte, and mesenchymal compartments, that regulate hair growth, pigmentation, and follicular remodeling [59]. Among these, mesenchymal stem cells (MSCs) contribute to follicular inductivity and pigmentation by supporting epithelial–mesenchymal signaling networks essential for hair cycling [60,61]. In parallel, dermal MSCs are involved in cutaneous repair and promote wound healing, extracellular matrix remodeling, and attenuation of inflammation in skin lesions [62,63].

A key feature of these stem cell populations is their sensitivity to redox balance. Hair-follicle-derived MSCs exhibit pronounced susceptibility to oxidative injury, with ROS accumulation driving apoptosis and functional decline [64]. This vulnerability becomes especially relevant in translational contexts, where ex vivo expansion and transplantation expose MSCs to oxidative environments that can significantly diminish their functional utility in clinical applications. Together, these observations highlight redox regulation as a key determinant of stem cell function in skin and hair biology and show that antioxidant systems are critical modulators of regenerative outcomes. Given lactoferrin’s antioxidant and regenerative potential, here we review specific in vitro studies that are important for predicting its potential clinical benefits.

An in vitro study has shown that lactoferrin is able to protect MSCs from oxidative-stress-induced cellular senescence (aging) and programmed cell death (apoptosis) [56]. Lactoferrin was able to significantly reduce the production of intracellular ROS in hMSCs induced by hydrogen peroxide exposure, restore growth, and promote self-renewal. Mechanistically, this was linked to inhibition of caspase-3 activation, a key enzyme driving apoptosis, and modulation of Akt signaling, involved in cell survival pathways [56]. These data highlight the capacity of lactoferrin as an antioxidant in mesenchymal cells, helping to limit ROS-induced damage at the cellular level. This positions lactoferrin as a protective and anti-senescence agent in contexts where oxidative stress limits regenerative capacity, such as in hair and skin development and repair.

Essential to our understanding of hair growth and repair are studies in dermal papilla (DP) cells, specialized mesenchymal cells that sit at the control center of the hair follicle. DP cells have regenerative properties, are associated with stem cells, and are critical for hair development and hair cycling [65]. They are often used in experimental settings to study the molecular basis of hair growth interventions [66]. One such study focused on colostrum-derived exosomes containing lactoferrin and proposed them as a cell-free regenerative factor that can increase DP cell proliferation and stimulate cell cycle progression [67]. A key biological switch controlling hair regeneration is the transition of the hair follicle from the telogen (resting) phase into the anagen (growth) phase. In mice, these exosomes were able to accelerate the transition from the telogen to the anagen phase via strong activation of the Wnt/β-catenin signaling pathway [67]. Thus, this cell-free system triggered a key regenerative hair switch in vivo, acting through the Wnt pathway to regulate regeneration.

Another study showed that lactoferrin can significantly increase the proliferation of cultured DP cells via modulation of growth-factor signaling [57]. Treatment with bovine lactoferrin (bLF) enhanced the phosphorylation of Erk1/2 and Akt in DP cells, important factors for survival and proliferation. Lactoferrin also induced the expression of several Wnt signaling-related components, including Wnt3a, Wnt7a, Lymphoid Enhancer-Binding Factor 1 (Lef1), and β-catenin [57]. This pathway plays a central role in hair follicle development, induction and cycling. Furthermore, topical application of lactoferrin in shaved mice significantly increased hair growth compared to controls, suggesting that bLF’s proliferative effects on DP cells in vitro can translate into enhanced hair growth potential in vivo [57].

Lactoferrin’s potential in cosmetic and skin care applications has been explored in skin keratinocytes and reconstructed epidermal models. Using a recombinant human lactoferrin from transgenic rice, research has shown that lactoferrin can activate growth and survival pathways in keratinocytes through the ERK/MAPK signaling pathway, mediated by the Low-Density Lipoprotein Receptor-Related Protein 1 (LRP-1) receptor [58]. Lactoferrin increased keratinocyte proliferation and motility, enhanced migration-associated cytoskeletal reorganization, and inhibited cell apoptosis. In a wound healing in vivo swine model, the protein promoted wound re-epithelialization by accelerating epidermal closure and overall wound healing rate following second-degree burn when applied topically [58]. These studies suggest that lactoferrin possesses healing and regeneration capabilities that can be leveraged in skin aging.

Recent work by Xie and colleagues using a reconstructed human epidermis (RHE) model provides compelling evidence for lactoferrin’s anti-inflammatory and barrier-supportive activity in epidermal tissue. In this three-dimensional keratinocyte-based system, lactoferrin reduced secretion of the pro-inflammatory markers IL-1α and Thymic Stromal Lymphopoietin (TSLP), while simultaneously improving the skin barrier as measured by increasing transepithelial electric resistance (TEER). Lactoferrin also upregulated skin barrier- and hydration-associated proteins, including involucrin, filaggrin, claudin-1, aquaporin-3, and hyaluronan synthase-1 [55]. These findings suggest that lactoferrin not only suppresses inflammatory signaling but also promotes barrier maturation within a physiologically relevant 3D skin model.

Studies across multiple epithelial cell types have consistently demonstrated lactoferrin’s ability to suppress inflammatory signaling and cytokine production. In intestinal epithelial models, lactoferrin treatment significantly reduced pro-inflammatory cytokine expression, including IL-1β, IL-6, TNF-α, and IL-8, while simultaneously downregulating NK-kB phosphorylation associated with MAPK signaling pathways [68,69,70]. A study in primary intestinal epithelial cells further revealed that lactoferrin’s anti-inflammatory effects involve modulation of the ELAVL1/PI3K/NF–κB pathway, with iron-free lactoferrin showing potent activity in reducing LPS-induced inflammatory responses [71]. Similar suppressive effects have been observed in bovine mammary epithelial cells, where lactoferrin pretreatment inhibited TLR4-mediated NF–κB activation and reduced expression of key inflammatory mediators [72]. Collectively, these studies establish lactoferrin as a key regulator of epithelial immune responses.

Unlike corticosteroids, which broadly suppress inflammatory gene transcription through glucocorticoid receptor signaling [73], lactoferrin is a systems-level microenvironment regulator rather than a generalized immunosuppressive agent. A comprehensive meta-analysis of lactoferrin studies demonstrated significant inhibitory effects on both upstream inflammatory cytokines and downstream NF–κB signaling across multiple epithelial models [7]. Contrary to general anti-inflammatory agents, lactoferrin exerts more moderate context-dependent immunomodulatory effects while maintaining tissue homeostasis at host-environment interfaces. Overall, lactoferrin functions as a key regulator of the epithelial microenvironment, coordinating antimicrobial defense, inflammatory balance, and oxidative stress control.

## 5. Lactoferrin Clinical Applications in Dermatology

There are limited studies currently exploring lactoferrin as an oral or topical formulation. No registered clinical trials have evaluated lactoferrin for hair-related conditions. However, evidence from in vitro and in vivo models suggests that lactoferrin may have the potential to mitigate hair loss or graying [57,74,75]. By contrast, clinical evidence exists exploring oral or topical lactoferrin in the context of acne and skin wound healing, with emerging data on broader inflammatory skin responses [76]. Studies conducted between 2010 and 2022 have reported that lactoferrin, when administered orally, can significantly reduce acne severity (Table 1) [77,78,79,80]. Results are indicated in the table below.

Collectively, these clinical studies suggest that lactoferrin can reach the skin target, modulate inflammation, and exert clinically measurable effects within epithelial tissues following oral administration. This supports lactoferrin’s potential as a supportive agent in the field of dermatology.

## 6. Future Directions

Current and past research in dermatology and cosmetics using lactoferrin has primarily focused on cell lines, animal models, and, in one case, reconstructed skin [55]. Limited clinical studies exist (on skin applications only), and ex vivo studies using explanted hair follicles or human skin are currently lacking.

Ex vivo explants represent the closest model to clinical testing and provide invaluable insights into optimal safe dosage, ensuring no toxicity, and accurate MoA. These data are empirical prior to initiating clinical trials, where MoA and dosage determine inclusion criteria, treatment regimens, and relevant endpoints. In addition, explants are particularly valuable in cosmetic and dermatology research, where increasing regulatory limitations on preclinical animal testing make alternative methods essential.

Ex vivo models have been previously established and validated in the literature for both hair follicles [81] and skin explants [82,83], providing reliable platforms for studying follicular and dermatological processes. Building on this established foundation, we propose to leverage these well-characterized systems to evaluate lactoferrin’s potential in hair and skin health. Studies would be conducted in specialized laboratories with access to clinical samples and established expertise in hair follicular and skin biology. The experimental approach would compare lactoferrin, with or without its peptide derivatives, using hypoxic versus normoxic skin and hair follicle explants. This design would help to identify safe and optimal dosing parameters ahead of clinical trials, while better defining lactoferrin’s MoA.

Following ex vivo validation, subsequent clinical studies could be conducted in a defined target population over a predetermined time frame, with endpoints tailored to the specific research objectives. The formulation vehicle for lactoferrin, whether topical or ingestible, would be selected and optimized for each treatment approach. A double-blind, controlled trial design would be preferred, particularly for studies involving oral lactoferrin administration.

One current limitation is the relatively small number of well-controlled clinical studies aimed at defining optimal effective dosing strategies for lactoferrin in distinct applications, such as skin health or hair support. Future studies focusing on evaluating comprehensive dose–response characterization in humans, along with standardized dosing regimens and long-term outcomes, will be important to better understand lactoferrin as a dietary supplement for hair and skin applications.

## 7. Conclusions on Lactoferrin in Hair and Skin Regeneration

This review provides an in-depth discussion about the role of lactoferrin and similar molecules in skin and hair applications and explores their potential use in nutraceutical formulations, with a special focus on hair growth and skin regeneration. Advances in recombinant protein production, including precision fermentation-derived human lactoferrin, further expand its potential as a scalable and biologically relevant active ingredient. Distinct from drug-like agents, lactoferrin is an endogenously produced balanced regulator of iron homeostasis, inflammation, and epithelial regeneration. Lactoferrin is not the most potent agent in any single category, but based on its mechanism of action, it can deliver coordinated multi-pathway improvements with potential to collectively outperform single-mechanism agents in complex epithelial environments.

Due to its high iron-binding capacity, lactoferrin and its derivatives can modulate iron levels in the extracellular environment. It can limit pathogen growth and protect from oxidative stress by sequestering excess free iron, and depending on the local microenvironment, it can facilitate the controlled delivery of iron to the cells required for metabolism and proliferation. This dual capacity of lactoferrin makes it an ideal candidate for skin and hair applications.

In several experimental models, lactoferrin and its derivatives have demonstrated the ability to protect against oxidative stress through both direct (ROS scavenging and chelation) and indirect (receptor binding) MoA (Figure 1). They also have the capacity to reduce pro-inflammatory molecules involved in chronic low-grade inflammation (“inflammaging”) and auto-immune responses. Additionally, lactoferrin exhibits regenerative properties, either by directly binding to the LRP1 receptor or indirectly through HIF-1 stabilization.

Ex vivo studies combined with targeted clinical trials can establish lactoferrin as a safe and effective nutritional supplement for skin and hair applications. Available research demonstrates encouraging consistency across studies evaluating inflammatory skin conditions, particularly acne [77,78,79,80]. Across acne-focused clinical studies conducted between 2010 and 2022, oral lactoferrin supplementation was associated with improvements in inflammatory lesion counts, acne severity, sebum production, and overall skin appearance, with reporter reductions in inflammatory lesions ranging from approximately 20–40% in placebo-controlled settings. Although formulations, dosing strategies, and study designs varied, findings were directly consistent and collectively suggest that orally administered lactoferrin can exert biologically relevant effects in the skin.

In contrast, no registered clinical trials have specifically evaluated lactoferrin for hair-related conditions, despite emerging mechanistic and preclinical evidence supporting potential relevance in follicular biology, oxidative stress regulation, and inflammatory modulation. While direct clinical evidence in AGA and AA is currently lacking, the high vascularization and immunological activity of the hair follicle support its potential as a target for circulating lactoferrin, warranting further investigation.

Importantly, the observed improvements in acne outcomes support broader mechanistic concepts relevant to skin and hair biology, including modulation of inflammatory signaling, oxidative stress, microbial interactions, and epithelial homeostasis. Based on the current available preclinical and clinical literature, a daily dose of 100–200 mg lactoferrin is a reasonable recommended supplementation range supporting intended biological applications. However, as mentioned above, further dose–response and long-term clinical studies are needed to optimize dosing strategies according to specific health outcomes. In conclusion, lactoferrin represents a promising dietary supplement for hair and skin conditions, and future studies will provide a critical foundation for lactoferrin’s rational use in the fields of dermatology and cosmetics.

## Figures and Tables

**Table 1:** Summary of human clinical trials (2010–2022) exploring lactoferrin supplementation for acne severity reduction.

Study	n	Age/ Gender	Design	Intervention	Duration	Clinical Outcome
Kim et al., 2010 [78]	36 (18 per group)	18–30 years; mixed gender	Randomized placebo-controlled study	200 mg of lactoferrin daily in fermented milk	12 weeks	Significant multi-parameter improvements Inflammatory lesion count (38.6% lower), total lesion count (23.1% lower), acne grade (20.3% lower, and sebum content (31.1% lower)
Mueller et al., 2011 [79]	43 (39 completed)	Adolescents and young adults; mixed gender	Open-label, single-arm study	100 mg of lactoferrin administered twice daily in a chewable form	8 weeks	76.9% of subjects showed improvement Reductions in total acne lesions and inflammatory activity
Chan et al., 2017 [80]	168 (164 completed)	13–40 years; mixed gender	Randomized, double-blinded, placebo-controlled study	100 mg lactoferrin (2× daily) Capsule form with 11 IU vitamin D and 5 mg zinc	12 weeks	32.5% reduction in acne lesions Maximal effects at week 10. Significant reduction in comedones and inflammatory lesions (44%) plus sebum secretion (6.2%) after 12 weeks compared to the placebo group
Kazandjieva et al., 2022 [77]	184	25–40 years; female only	Randomized, assessor-blinded, parallel group, multicenter trial	Topical cream + oral supplement Sebogard Elle cream (2× daily) plus daily oral prebiotic with fructose/galactose oligosaccharides, zinc, lactoferrin, and niacinamide, vs. cream alone	12 weeks	Superior combination therapy results Combination group: 73% reduction in inflammatory acne lesions vs. topical only group: 63% reduction

Table 1 summarizes human clinical trials between 2010 and 2022 exploring lactoferrin as a supplement to reduce the severity of acne.

## Data Availability

No new data were created or analyzed in this study. Data sharing is not applicable to this article.

## Abbreviations

The following abbreviations are used in this manuscript:

AA	Alopecia Areata
AGA	Androgenic alopecia
AKT	v-akt murine thymoma viral oncogene homolog
DP	Dermal papilla
ERK	Extracellular signal-regulated kinase
HIF-1	Hypoxia- inducible factor-1
IL-6	Interleukin 6
LRP1	Low-Density Lipoprotein Receptor-Related Protein 1
MAPK	Mitogen-activated protein kinase
MoA	Mechanism of action
MSC	Mesenchymal stem cells
NFkB	Nuclear factor kappa light chain enhancer of activated B cells
PHD-2	Prolyl-hydroxylase-2
ROS	Reactive oxygen species
TE	Telogen effluvium
TNFα	Tumor necrosis factor alpha
UV	Ultraviolet
